# ST-segment elevation in patients presenting with COVID-19: case series

**DOI:** 10.1093/ehjcr/ytaa553

**Published:** 2021-02-08

**Authors:** Mehrdad Saririan, Richard Armstrong, Jon C George, Bartosz Olechowski, Stephen O’Connor, James Brian Byrd, Andrew R Chapman

**Affiliations:** 1 Division of Cardiology, Valleywise Health/Creighton University, Phoenix, AZ, USA; 2 Department of Cardiology, St James’s Hospital Dublin, Republic of Ireland; 3 Division of Interventional Cardiology, Einstein Medical Center, Philadelphia, PA, USA; 4 Dorset Heart Centre, Royal Bournemouth & Christchurch Hospitals NHS Foundation Trust Bournemouth, UK; 5 Division of Cardiovascular Medicine, University of Michigan Medical School, Ann Arbor, MI, USA; 6 BHF Centre for Cardiovascular Science, University of Edinburgh, Chancellors Building, Royal Infirmary of Edinburgh, Edinburgh EH16 4SA, UK

**Keywords:** COVID-19, STEMI, Histology, Case series, Case report

## Abstract

**Background:**

The severe acute respiratory syndrome coronavirus 2 (SARS-CoV-2) is the pathogen responsible for the now pandemic disease, coronavirus disease (COVID-19). A number of reports have emerged suggesting these patients may present with signs and symptoms consistent with ST-segment elevation myocardial infarction without coronary artery occlusion.

**Case summary:**

We report an international case series of patients with confirmed COVID-19 infection who presented with suspected ST-segment elevation myocardial infarction. Three patients with confirmed COVID-19 presented with electrocardiogram criteria for ST-segment elevation myocardial infarction. No patient had obstructive coronary disease at coronary angiography. Post-mortem histology in one case demonstrated myocardial ischaemia in the absence of coronary atherothrombosis or myocarditis.

**Discussion:**

Patients with COVID-19 may present with features consistent with ST-segment elevation myocardial infarction and patent coronary arteries. The prevalence and clinical outcomes of this condition require systematic investigation in consecutive unselected patients.

**Learning points**

- Patients with coronavirus disease (COVID-19) may present with ST-segment elevation, where urgent coronary angiography should be considered in line with established international guidance.
- In this series, three patients presenting with ST-segment elevation on the electrocardiogram, which may have been consistent with myocardial infarction, had unobstructed coronary arteries at coronary angiography.
- Studies of consecutive patients with COVID-19 are necessary to identify the true prevalence of ST-segment elevation and myocardial injury on biomarker testing.

## Introduction

The severe acute respiratory syndrome coronavirus 2 (SARS-CoV-2) is a novel pathogen responsible for the pandemic disease, coronavirus disease (COVID-19). This is most often a respiratory illness, which can be asymptomatic, cause mild upper respiratory tract symptoms, or result in severe bilateral pneumonia, acute respiratory distress syndrome, and death. In hospitalized patients with COVID-19, myocardial injury is observed in between 23% and 27.8% of cases.^1^^,^^2^

Here, we describe a series of patients with COVID-19 who presented to three international centres with ST-segment elevation, and discuss their presenting features, clinical findings, and outcomes.

The usefulness of a paper describing electrocardiogram (ECG) changes suggestive of ST-segment elevation myocardial infarction in the context of COVID-19 infection was identified when authors noted discussion of such cases on social media. Two authors suggested this possibility on Twitter and were contacted by the authors who contributed cases. We included cases in which patients had confirmed SARS-CoV-2 infection on viral polymerase chain reaction, and excluded those who were negative on testing or where coronary angiography was not undertaken. Informed consent was obtained from patients or their relatives where appropriate. Formal Institutional Review Board approval was not required from any participating centre.

## Timeline

**Figure:**
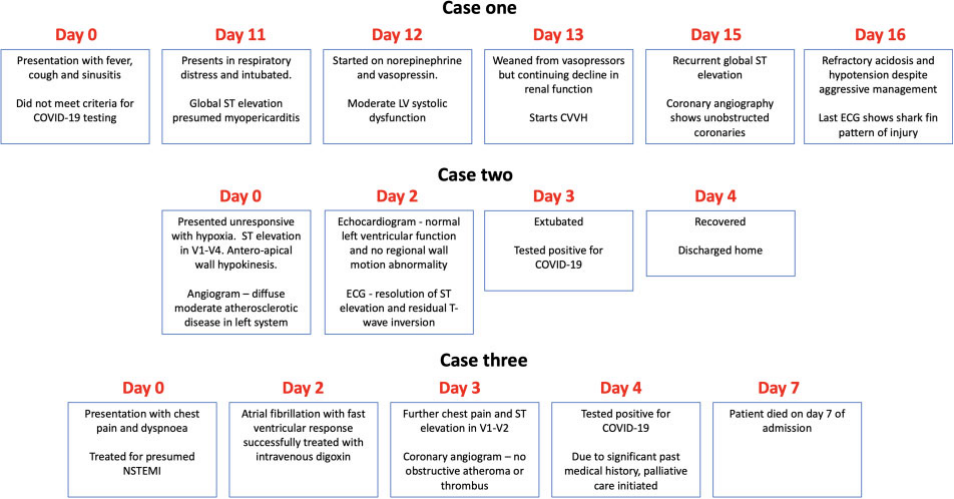


## Case presentation

### Case 1

**Figure 1:**
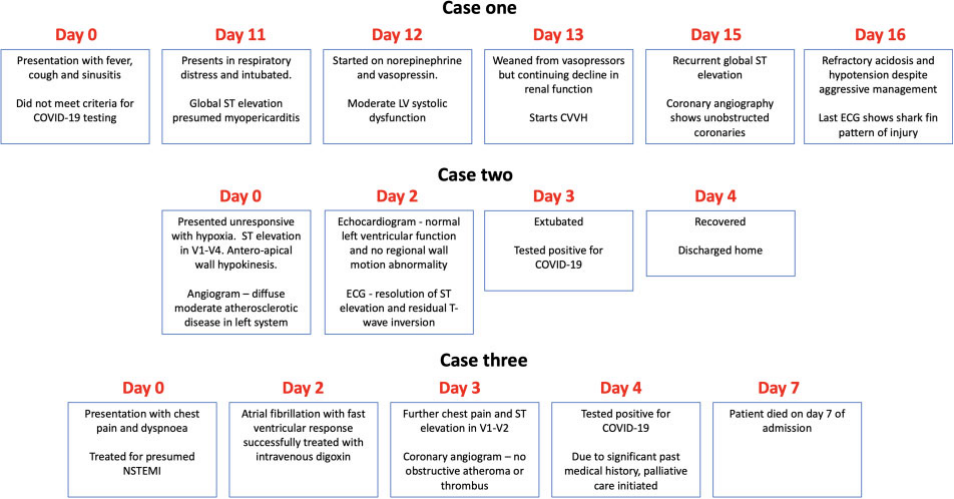
Key timepoints during each case illness.

A 61-year-old male presented to the Emergency Department with generalized body aches, fever, and worsening cough (*Figure 1*). He recently started antibiotics for sinusitis but denied any known contact with persons infected with SARS-CoV-2. His medical history was notable for hypertension and diet-controlled diabetes. His only medications were lisinopril and aspirin. At presentation, he was febrile at 39.3°C and his oxygen saturations were 95%. The remainder of his physical exam was normal, as were his labs, chest X-ray, and ECG. The patient did not meet the established criteria for SARS-CoV-2 testing and he was discharged with strict instructions to self-quarantine.

On Day 11, the patient returned to the hospital with shortness of breath. There was no chest pain. He was in respiratory distress with oxygen saturations of 85% on 15 L/min. He was immediately moved to a negative-pressure isolation room and was intubated. Lymphopenia (1%; normal range 14.6–47.9%) was present. His arterial blood gas after intubation on 100% FiO_2_ demonstrated a pH of 7.28, P0_2_ 200 mmHg, PCO_2_ 38.4 mmHg, HCO3 18 mmol/L, and Lactate of 4.1 mmol/L. Shortly after intubation, he developed supraventricular tachycardia at a rate of 198 b.p.m. (Supplementary material online, *Figure S1*) which was successfully treated with intravenous (IV) adenosine. The post-conversion ECG showed 2 mm of antero-lateral ST-elevation without reciprocal depression (Supplementary material online, *Figure S2*). Initial troponin I was 6283 ng/L (normal range <40 ng/L). Chest X-ray showed new bilateral airspace opacities (Supplementary material online, *Figure S3*). The on-call interventional cardiologist suspected myopericarditis and deferred immediate angiography.

**Figure 2:**
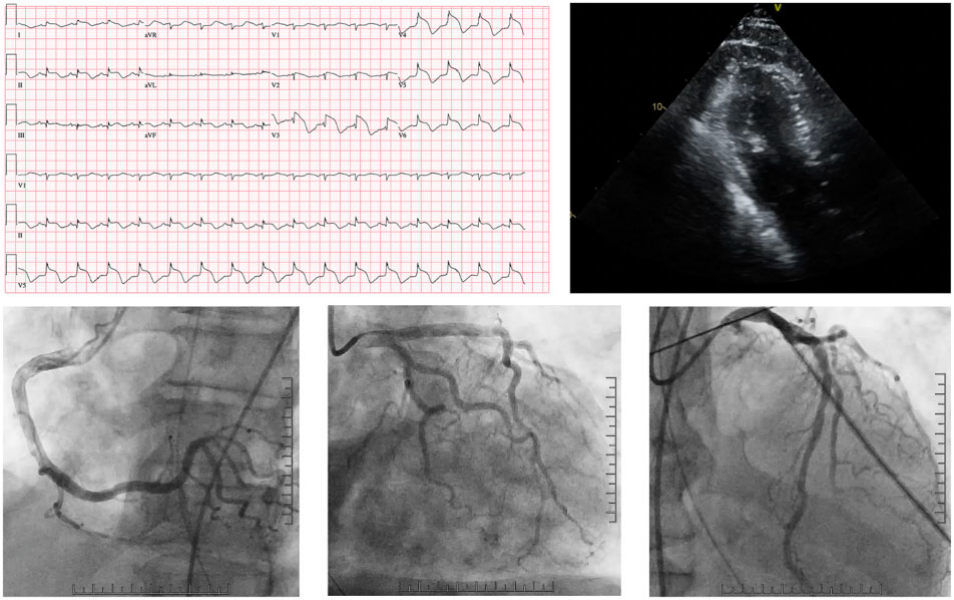
Lab activating electrocardiogram, echocardiogram, and coronary angiography findings in Case 1.

The patient became progressively hypotensive and was started on high-dose norepinephrine and vasopressin with empirical ceftriaxone, azithromycin, and hydroxychloroquine. The patient was given a loading dose of ticagrelor and IV heparin. The troponin was 7457 ng/L 3 h after the first ECG, and by 12 h had decreased to 5852 ng/L. A transthoracic echocardiogram showed moderate left ventricular systolic dysfunction (*Figure 2*). The serum troponin continued to fall to 2159 ng/L, and by Day 12, the patient had been weaned from vasopressors. However, his renal function rapidly deteriorated (baseline creatinine 0.89 mg/dL, peak 6.19 mg/dL, normal range 0.84–1.21 mg/dL), such that by Day 13, he was anuric and continuous veno-venous hemofiltration (CVVH) was started.

A repeat ECG on Day 15 showed recurrent global ST-elevation pattern (*Figure 2*). Coronary angiography was undertaken which revealed no luminal stenosis or thrombosis, with preserved TIMI 3 flow in all coronary arteries (*Movies 1–3*). Left ventriculography revealed mild apical hypokinesis (Supplementary material online, *Movie S4*). The patient was returned to the intensive care unit (ICU) with a presumptive diagnosis of COVID-19 associated myocarditis, and IV solumedrol and intravenous immunoglobulin (IVIG) were started. His troponin continued to decrease, last measured at 768 ng/L.

**Figure 3:**
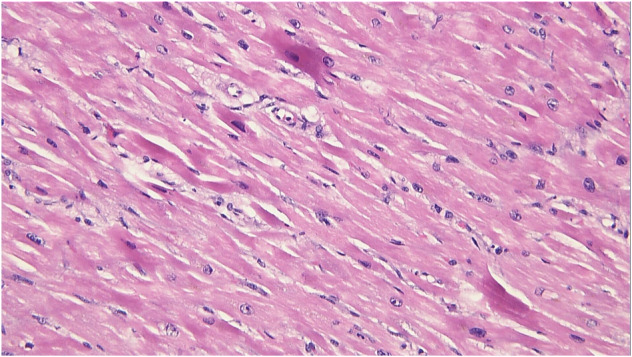
Histology from left ventricular section taken at post-mortem in Case 1.

Despite aggressive supportive measures, the patient became progressively hypotensive and acidotic. His final ECG demonstrated a dramatic shark fin pattern indicative of a diffuse myocardial process (Supplementary material online, *Figure S4*) and shortly thereafter he went into cardiac arrest (pulseless electrical activity). He was on CVVH with a final potassium recorded at 6.0 mmol/L (normal range 3.5–5 mmol/L). COVID-19 infection was confirmed post-mortem, and an autopsy was performed. Left ventricular sections demonstrated focal eosin uptake indicative of myocardial ischaemia (*Figure 3*) with no evidence of atherothrombosis or myocarditis. These findings may be in keeping with type 2 myocardial infarction from profound myocardial oxygen supply mismatch in the context of significant refractory hypotension and acidaemia.

### Case 2

**Figure 4:**
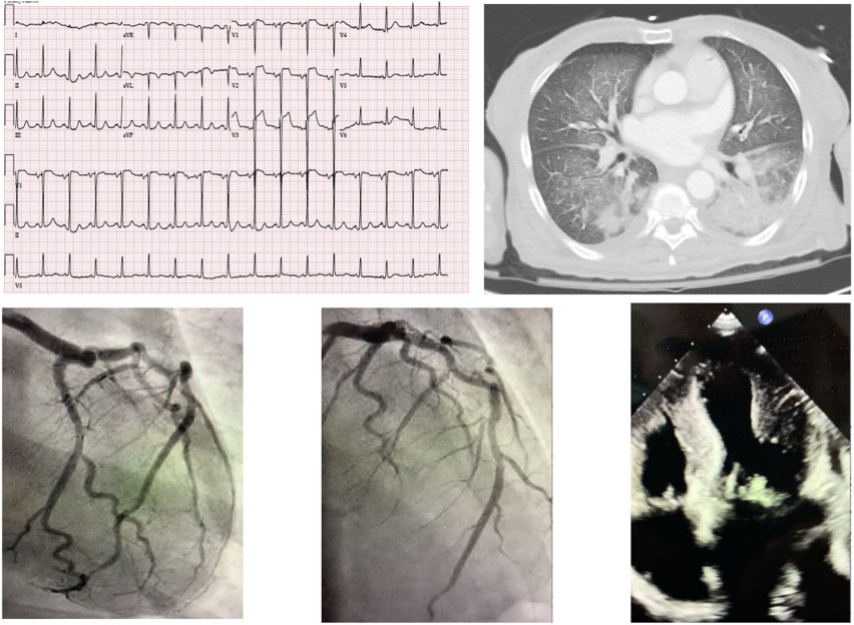
Lab activating electrocardiogram, computed tomography chest, echocardiogram, and coronary angiography findings in Case 2.

A 59-year-old female with a past medical history of chronic obstructive pulmonary disease and hypertension was brought in by ambulance after being found minimally responsive on the ground by neighbours. Her initial observations showed oxygen saturations of 80% on room air with altered mental status. She was intubated on arrival to the emergency department. Following intubation, the ECG (*Figure 4*) revealed ST-segment elevations in V1–V4 and reciprocal ST-depressions in leads II, III, and aVF. Due to concerns about COVID-19 and inability to obtain further medical history, computed tomography (CT) of the head, and chest was performed which revealed bilateral lower lung lobe infiltrates and pulmonary oedema with moderate calcification in the mid-left anterior descending artery (*Figure 3*). Bedside echocardiogram demonstrated reduced left ventricular ejection fraction of 40% with antero-apical wall hypokinesis. After discussion between the emergency department, cardiology, and ICU teams, a decision was made to perform coronary angiography. Moderate diffuse atherosclerotic disease was observed in the left system with no significant luminal obstruction elsewhere (*Figure 4* and Supplementary material online, *Movies S5* and *S6*) corresponding to the ECG findings. Left ventricular end-diastolic pressure was elevated at 30 mmHg.

The peak troponin I concentration was elevated at 2390 ng/L. A formal echocardiogram on Day 2 of hospitalization revealed normal left ventricular function with no significant wall motion abnormalities. A repeat ECG demonstrated resolution of the ST-segment elevation and residual T-wave inversion (Supplementary material online, *Figure S5*). The patient was extubated on Day 3 and tested positive for COVID-19. The attending clinician suspected myopericarditis. The patient was maintained in isolation for an additional day and as they were noted to be back at baseline, they were discharged home with instruction to self-quarantine for a further 14 days. Given the brisk improvement in ECG changes and the resolution of regional wall motion abnormality after correction of hypoxaemia, type 2 myocardial infarction seems a likely aetiology.

### Case 3

A 69-year-old female complained of acute onset chest tightness and dyspnoea. A 12-lead ECG revealed left bundle branch block which was known (Supplementary material online, *Figure S6*). She denied any recent travel and had no known exposure to SARS-CoV-2. She had a previous history of non-ischaemic heart failure with reduced ejection fraction and was on appropriately tailored heart failure therapy including bisoprolol, ramipril, spironolactone, and furosemide. Her baseline NYHA class was II, with a dry NT-proBNP of 899 ng/L (normal range <175 ng/L) 4 months previously. An implantable cardioverter-defibrillator was placed in 2004, however, given recovery in left ventricular function the device was not replaced when it reached end of life in 2018. Other background history was significant for motor neurone disease, diagnosed 4 years previously, and the patient required assistance in activities of daily living.

Initial assessment showed temperature 35.6°C, blood pressure 132/85, heart rate 103, oxygen saturations 87% on 4 L via nasal cannula and a respiratory rate of 33. Examination revealed reduced air entry at both lung bases. Chest X-ray revealed bilateral infiltrates (Supplementary material online, *Figure S7*). Blood panel revealed white cell count of 10.2 and lymphocytes of 4.4. Initial high-sensitivity troponin T concentrations were 51 ng/L, rising to 504 ng/L on serial testing. NT-proBNP was elevated at 16 857 ng/L. The patient was managed as presumed Non ST-segment elevation acute coronary syndrome (NSTE-ACS) with decompensated heart failure and loading dose dual antiplatelets, therapeutic low molecular weight heparin, high-dose IV diuretics, and IV nitrates were administered. On Day 2 of admission, a run of rapidly conducted atrial fibrillation was treated successfully with IV digoxin.

**Figure 5:**
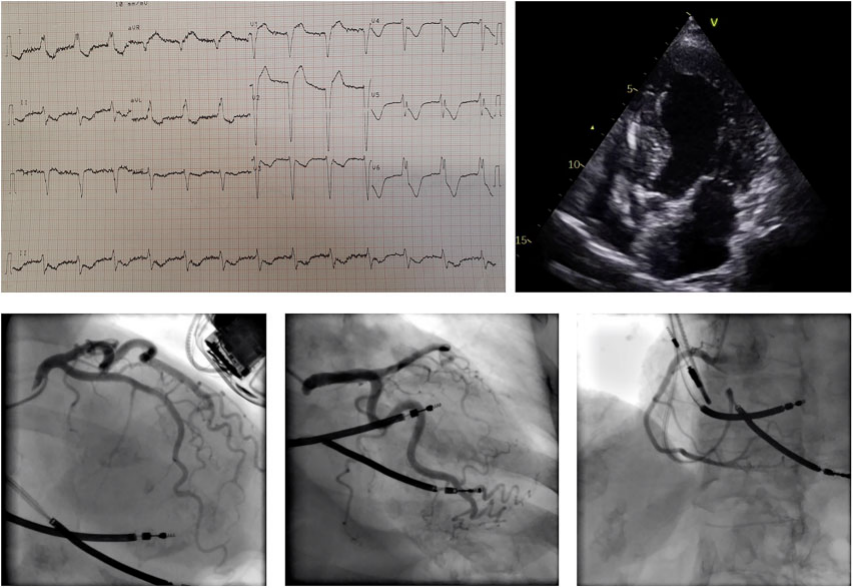
Lab activating electrocardiogram, echocardiogram, and coronary angiography findings in Case 3.

On Day 3 of admission, progressive dyspnoea, chest pain, hypotension, and oliguria developed and ECG changes were noted with progressive dynamic concordant ST-elevation in V1–V2 and ST-depression in V3–V5 (*Figure 5*). Bedside transthoracic echocardiography revealed impaired left ventricular function which was similar to baseline. The primary percutaneous coronary intervention pathway was activated and the patient emergently transferred. Coronary angiography was performed via a radial approach, which revealed no obstructive atheroma or thrombus (Supplementary material online, *Movies S7–S9*). The presumptive aetiology of her decompensated heart failure was type 2 myocardial infarction secondary to hypoxia and hypotension due to critical illness, or myocarditis. On Day 4 of her admission, SARS-CoV-2 swabs returned as positive. Given her history of motor neurone disease and progressive clinical decline, limits of treatment were discussed, and comfort measures instituted with help from palliative care colleagues. The patient died on Day 7 of admission.

## Discussion

This case series describes three patients who developed ST-segment elevation suspicious for myocardial infarction in the context of COVID-19 infection, who were found to have no angiographic evidence of atherothrombotic type 1 myocardial infarction. In all cases, type 2 myocardial infarction due to myocardial oxygen supply mismatch in the context of critical illness was felt to be the most likely aetiology. In one case, post-mortem examination of myocardial tissue at autopsy demonstrated evidence of ischaemia without thrombosis or myocarditis.

Myocardial injury has been described in up to 12% of hospitalized patients with COVID-19,^3^^,^^4^ and up to 20% of those requiring intensive care.^4^^,^^5^ One study suggested a high prevalence of abnormal findings on cardiac magnetic resonance imaging scans in patients with COVID-19 infection.^6^ Similarly, a global survey of echocardiography findings including 1272 patients with COVID-19 across 69 countries found abnormalities in almost half of all patients.^7^ However, all findings to date are susceptible to selection and reporting bias.

There are a number of plausible mechanisms for both direct and indirect myocardial injury due to COVID-19.^8^ Direct effects may be mediated by the SARS-CoV-2 virus harnessing angiotensin-converting enzyme-2 receptor to gain access into the host cell. Angiotensin-converting enzyme-2 is expressed within the myocardium and up-regulated in heart disease, and patients with cardiovascular disease may therefore be more vulnerable. This may lead to endothelial cell and microvascular dysfunction or occlusion.^9^ A recent case report from Bergamo, Italy, described evidence of coronary microthrombi at post-mortem in a patient with COVID-19 who presented with ST-segment elevation without obstructive coronary disease.^10^ This is consistent with reports of pulmonary microvascular thrombosis in patients with COVID-19,^11^ and diagnostic criteria for disseminated intravascular coagulation have been observed in 71.4% of non-survivors with COVID-19.^12^

COVID-19 and infection with other coronaviruses may lead to myocarditis.^13–15^ A recent report described lone COVID-19 myocarditis in a patient with no respiratory symptoms,^2^ and fulminant COVID-19-induced myocarditis has been described and treated with corticosteroids and human immunoglobulin.^13^ In addition, transfusion of convalescent plasma with an SARS-CoV-2–specific antibody (IgG) in addition to corticosteroid therapy has been described, leading to improvement in clinical state in a patient with myocardial injury.^16^ These treatments have not demonstrated efficacy in clinical trials.

Although histopathological characteristics of COVID-19 are similar to previously described coronaviruses causing SARS^17^ and Middle Eastern respiratory syndrome,^18^ pathological cardiac manifestations are poorly described. Whilst interstitial mononuclear inflammatory infiltrates have been observed at post-mortem in a patient with myocardial injury during their COVID-19 illness^19^ in our case, there was no evidence of myocarditis nor thrombosis.

It is possible the findings observed at autopsy are simply reflective of pathophysiological changes in type 2 myocardial infarction, which may have occurred due to profound refractory hypotension and acidosis, as is commonly observed in critically unwell patients requiring circulatory support.^20^ Indeed, all presented cases had clear evidence of myocardial oxygen supply or demand imbalance without evidence of atherothrombosis, satisfying the diagnostic criteria for type 2 myocardial infarction as per the Fourth Universal Definition of Myocardial Infarction.

In order to fully understand the mechanism of ST-elevation in patients with COVID-19, studies of consecutive patients who have undergone coronary angiography are required. In the largest case series of 18 patients, only 50% underwent coronary angiography.^21^ Where invasive coronary angiography is indicated, this should be undertaken with full personal protective equipment.^22^ Non-invasive imaging may aid diagnosis. The presence of a regional wall motion abnormality on echocardiography increases the likelihood of an acute atherothrombotic lesion and may lead clinicians to undertake coronary angiography. Conversely, the lack of regional changes may provide reassurance and lead to alternative non-invasive imaging methods such as CT coronary angiography or cardiac MRI.

As STEMI is more common in patients with recent respiratory infection,^23^ we would reiterate recommendations from the European Society of Cardiology Guidance for the Diagnosis and Management of Cardiovascular Disease during the COVID-19 pandemic, to promptly assess patients with ST-segment elevation in line with existing treatment protocols and consider urgent coronary angiography where acute coronary syndrome is suspected.^24^

There are several limitations to our analysis. This is a selected case series, and our findings cannot be generalized to all patients with COVID-19. However, in an evolving pandemic condition associated with significant morbidity and mortality, we believe it is important to consider these observations in the context of the emerging evidence base. Importantly, we identified but did not include four cases of possible STEMI in patients with clinically suspected COVID-19 as viral PCR swabs were negative for SARS-CoV-2. We would note the sensitivity for this test is reportedly as low as 70%, and where the clinical suspicion for COVID-19 is high, patients should be retested and isolated as appropriate.

## Conclusion

Patients with COVID-19 may present with ST-segment elevation suggestive of myocardial infarction in the absence of atherothrombosis. At present, the true prevalence of STEMI in COVID-19 cannot be determined and guidelines recommend we continue to approach all patients with ST-elevation with a high index of suspicion for coronary artery occlusion, to minimize delay to diagnosis and maximize treatment benefit.

## Lead author biography

**Figure:**
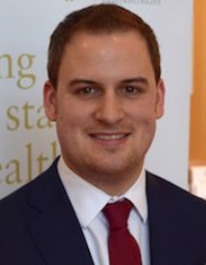


Dr Andrew R. Chapman is a Specialist Registrar and Clinical Lecturer in Cardiology at the Royal Infirmary of Edinburgh and University of Edinburgh, Scotland, UK.

## Supplementary material

Supplementary material is available at *European Heart Journal - Case Reports* online.

**Slide sets:** A fully edited slide set detailing this case and suitable for local presentation is available online as Supplementary data.

**Consent:** The authors confirm that written consent for submission and publication of this case series including images and associated text has been obtained from the patients in line with COPE guidance.

**Conflict of interest:** none declared.

**Funding:** A.R.C. is supported by a Starter Grant for Clinical Lecturers from the Academy of Medical Sciences [SGL021\1075]. There are no other funding declarations.

## Supplementary Material

ytaa553_Supplementary_Data
