# Supplementary material for: ST-segment elevation in patients presenting with COVID-19: case series
Source: Eur Heart J Case Rep. 2021 Feb 8;5(2):ytaa553. doi: 10.1093/ehjcr/ytaa553 (PMC7898565; doi:10.1093/ehjcr/ytaa553)
Supplement: ytaa553_Supplementary_Data [file ytaa553_supplementary_data.zip › RESUBMIT_Covid-STEMI-Supplement.docx]

SUPPLEMENTARY DATA

**Supplementary Figures: 6**

**Supplementary Movies: 18**

**Figure S1. SVT after intubation in Case One.**

**Figure S2. ST-segment elevation after cardioversion with Adenosine in Case One.**

**Figure S3. Chest X-Ray after intubation in Case One**


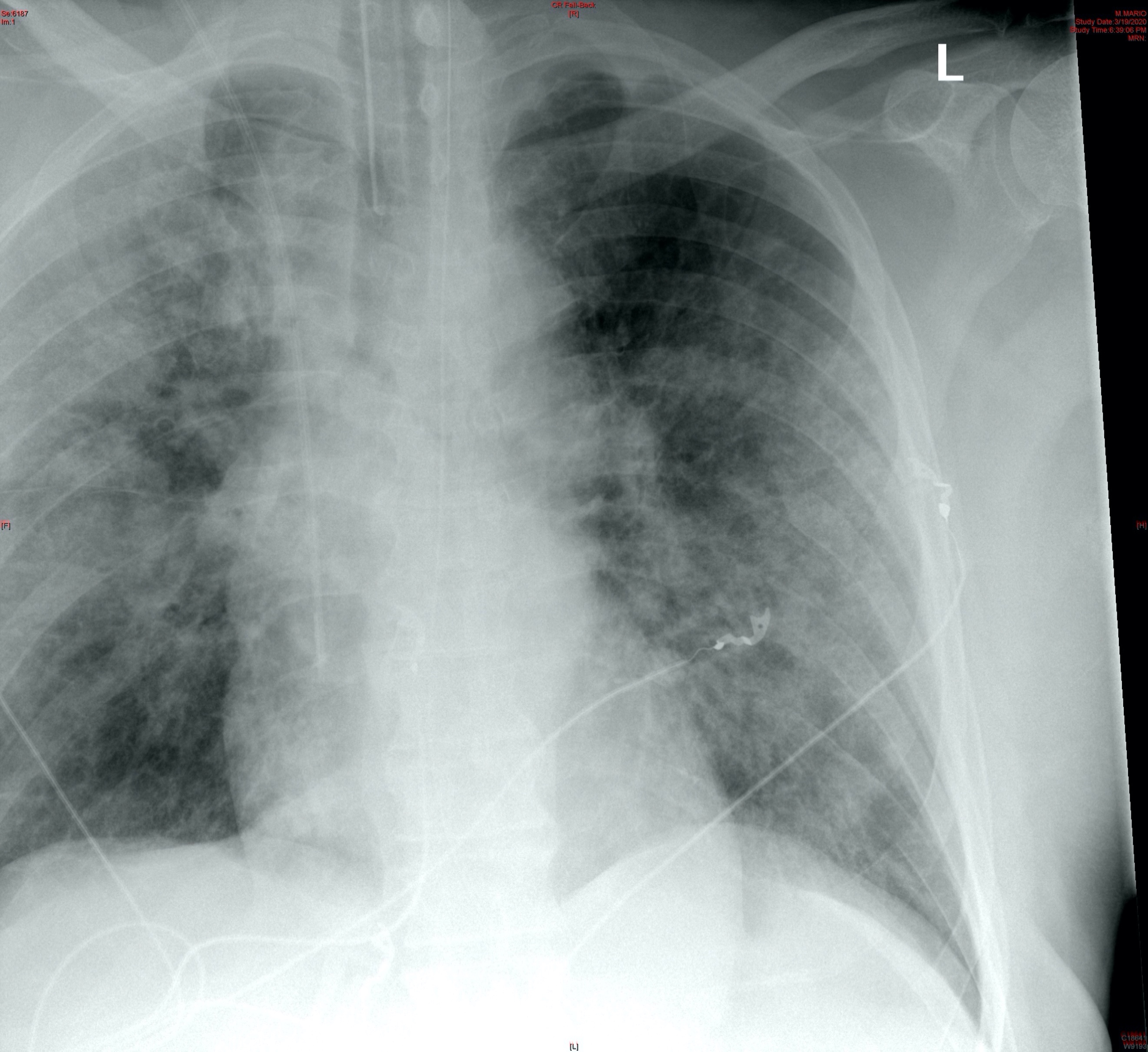


**Figure S4. Last recorded 12-lead ECG in Case One.**

**Figure S5. Resolution of ST elevation in Case Two.**
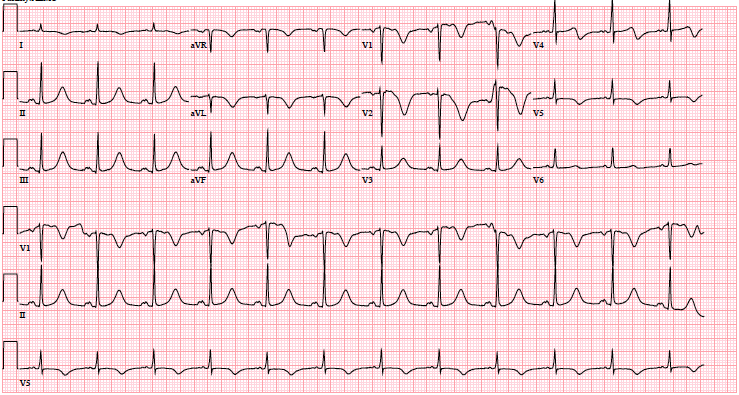


**Figure S6. Presenting ECG in Case Three**

**
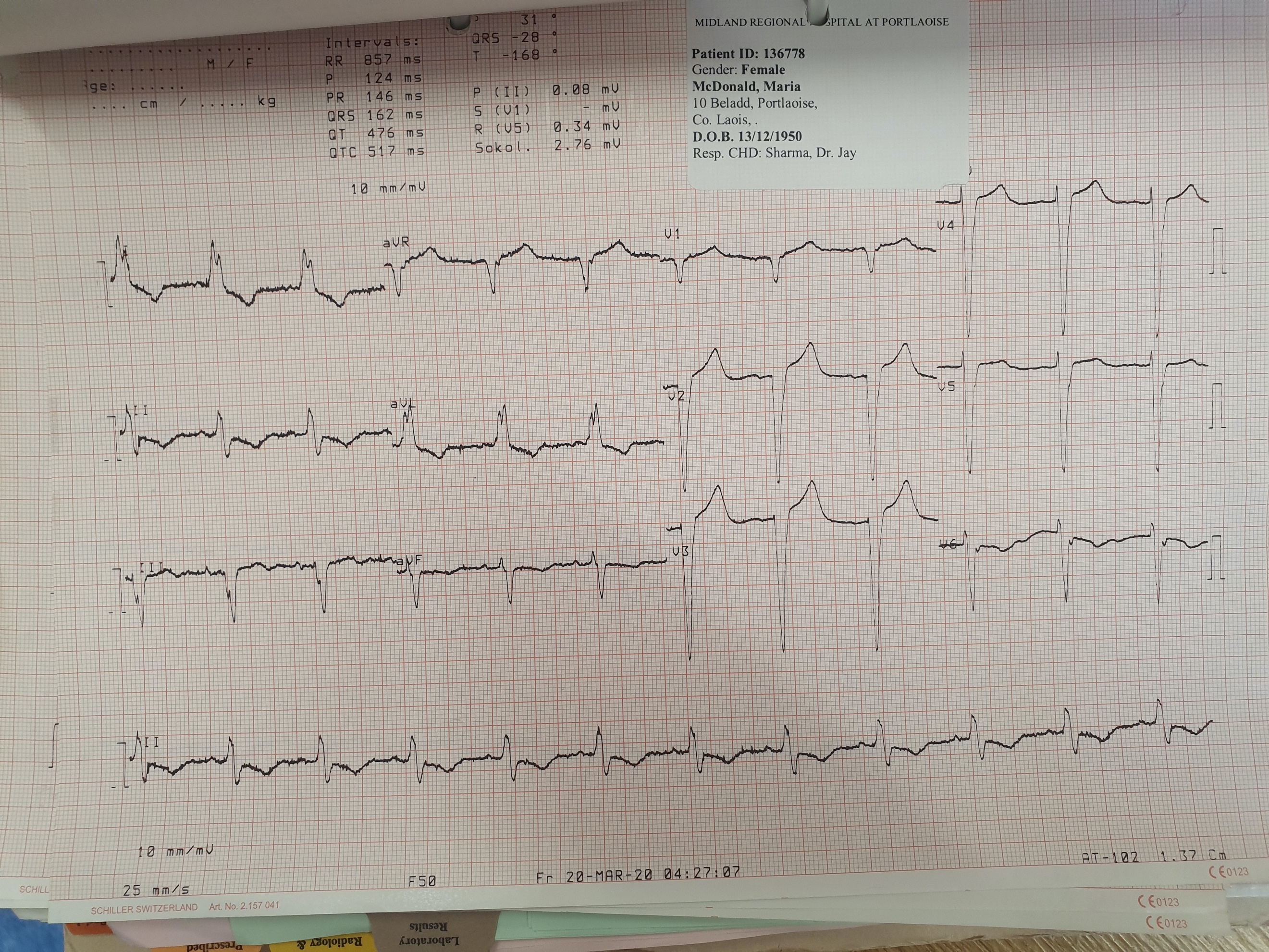
**

**Figure S7. Chest X-Ray on admission in Case Three.**

**
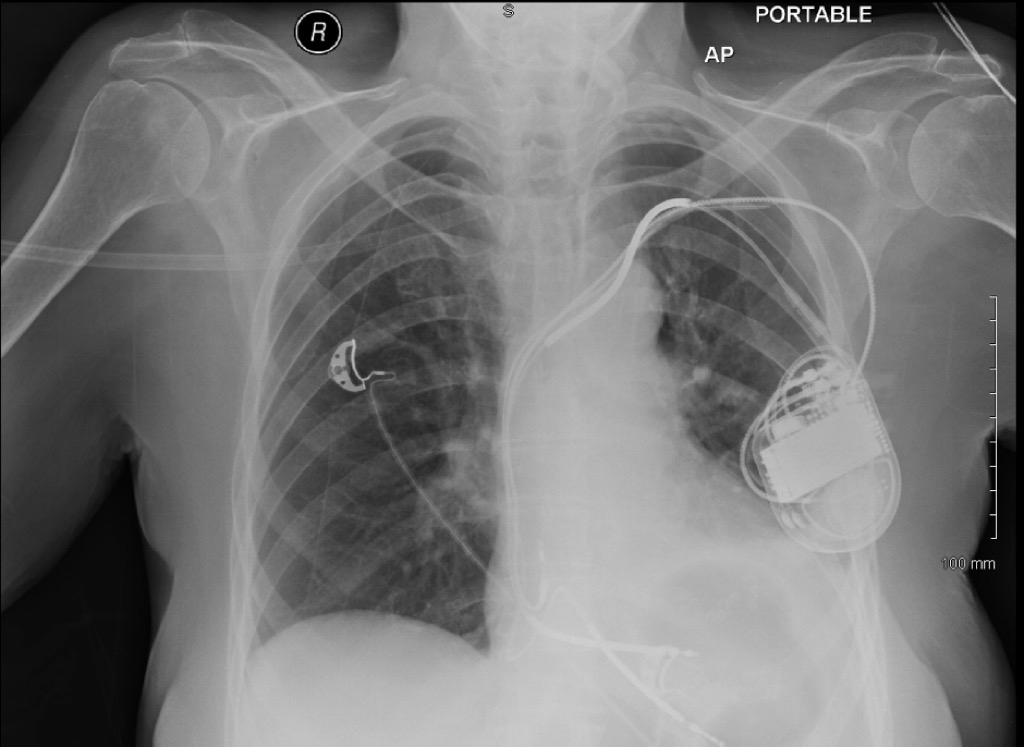
**

**Supplementary Movie 1. Left anterior descending coronary angiography in case one**

**Supplementary Movie 2. Left circumflex coronary angiography in case one**

**Supplementary Movie 3. Right coronary artery coronary angiography in case one**

**Supplementary Movie 4. Left ventriculogram in case one**

**Supplementary Movie 5. Left anterior descending coronary angiography in case two**

**Supplementary Movie 6. Left circumflex coronary angiography in case two**

**Supplementary Movie 7. Left anterior descending coronary angiography in case three**

**Supplementary Movie 8. Left circumflex coronary angiography in case three**

**Supplementary Movie 9. Right coronary artery coronary angiography in case three**
